# Non-Invasive and Long-Term Electrophysiological Monitoring Sensors for Cerebral Organoids Differentiation

**DOI:** 10.3390/bios15030173

**Published:** 2025-03-07

**Authors:** Yan Jin, Yixun Guo, Qiushi Li, Lei Wu, Yuqing Ge, Jianlong Zhao

**Affiliations:** 1 College of Sciences, Shanghai Institute of Technology, Shanghai 201418, China; jinyan@sit.edu.cn (Y.J.); 226181134@mail.sit.edu.cn (Y.G.); 2 State Key Laboratory of Transducer Technology, Shanghai Institute of Microsystem and Information Technology, Chinese Academy of Sciences, Shanghai 200050, China; liqiushi@mail.sim.ac.cn (Q.L.); wulei@mail.sim.ac.cn (L.W.); 3 Center of Materials Science and Optoelectronics Engineering, University of Chinese Academy of Sciences, Beijing 100049, China; 4 Shanghai Frontier Innovation Research Institute, Shanghai 201108, China

**Keywords:** microelectrode array, electrophysiological activities, cerebral organoids, Pt black

## Abstract

Cerebral organoids derived from human induced pluripotent stem cells (iPSCs) have emerged as powerful in vitro models for studying human brain development and neurological disorders. Understanding the electrophysiological properties of these organoids is crucial for evaluating their functional maturity and potential applications. However, the differentiation and maturation of stem cells into cerebral organoids is a long, slow, and error-prone process. Hence, it is vitally crucial to establish a non-invasive method of monitoring the process over a long period of time. In this study, a planar microelectrode array (MEA) with platinum (Pt) black electroplating is designed to monitor the electrophysiological activities and pharmacological responses of cerebral organoids using an external neural signal acquisition system interfaced with the MEA. The planar MEA with Pt black electroplating has a significantly reduced electrode impedance and exhibits a robust capability for the real-time detection of spontaneous neural activities, including extracellular spikes and local field potentials. Distinct electrophysiological signal strengths in cerebral organoids were observed at early and late developmental stages. Further pharmacological stimulations showed that 30 mM KCl would induce a marked increase in spike rate, indicating an enhancement of neuronal depolarization and an elevation of network excitability. This robust response to KCl stimulation in mature networks serves as a reliable indicator of neural maturity in cerebral organoids and underscores the platform’s potential for drug screening applications. This work highlights the integration of MEA technology with cerebral organoids, offering a powerful platform for real-time electrophysiological monitoring. It provides new insights into the functional maturation of neural networks and establishes a reliable system for drug screening and disease modeling, facilitating future research into human brain physiology and pathology.

## 1. Introduction

Due to the practical and ethical constraints associated with direct research on the human brain, along with the limitations imposed by interspecies differences in animal models, in vitro models utilizing human cells have emerged as a compelling alternative for investigating neuronal circuitry, neurotoxicity, neurological disorders, and brain development [1,2,3]. Advancements in the understanding of human brain development increasingly rely on biosystems generated from three-dimensional (3D) neural cultures, including cortical spheroids, organoids, and assembloids [4,5,6,7]. These 3D structures are cultured in vitro from induced pluripotent stem cells (iPSCs) or embryonic stem cells (ESCs) and effectively simulate the developmental processes of the human brain and various neuronal subtypes [8,9].

The cerebral organoids developed as models for neural development represent a significant advancement in the field of brain research [10,11]. These structures overcome the limitations of traditional 2D neural cultures such as the lack of supporting cell types and spatial structure, offering a closer approximation of the complexity of the human brain [12,13]. The advent of cerebral organoids not only provides a novel platform for studying the fundamental biology of the nervous system but also demonstrates significant potential for modeling complex neurological diseases, such as Alzheimer’s disease, Parkinson’s disease, and autism [14,15,16]. Despite their remarkable potential, challenges including reproducibility, long-term viability, and accuracy in terms of simulating advanced brain regions remain focal points for the ongoing research [17,18,19]. These challenges highlight the critical role of electrophysiological characterization in assessing the functional maturity of cerebral organoids. Unlike molecular or structural analyses, electrophysiological-biosensor directly evaluates neural activity and connectivity through synaptic potentials generated by neurons, providing essential insights into the dynamic functionality of organoid-derived neural networks [20].

As electrophysiological studies facilitate the observation of spontaneous firing and synchronized activities of neurons, providing valuable insights into the complex neural circuits within the brain, accurate monitoring of the electrophysiological characteristics of these 3D organoids can enhance the understanding of the interactions involved in the development of the nervous system, as well as the evolution and origins of abnormal behaviors and disease conditions. At present, the electrophysiological assessment for organoids is still challenging, mainly focused on obtaining stable signal stability in the organoid’s complex 3D structure and achieving high spatial resolution to capture fine-scale neural interactions, which are crucial for advancing the fidelity of organoid-based neural models. Microelectrode arrays (MEA) have emerged as a powerful tool for electrophysiological monitoring of neuronal activity [21], offering several advantages over conventional techniques like patch-clamp and calcium imaging. Unlike patch-clamp, which is labor-intensive and limited to single-cell recordings [22,23], MEA allows for high-throughput, multi-channel monitoring of neural networks. While calcium imaging provides valuable insights into neural activity [24,25], it lacks the temporal resolution required to detect action potentials. In contrast, MEA can capture both spontaneous firing and synchronized activity, providing a more comprehensive view of network function. However, challenges remain in signal stability and sensitivity, particularly in complex 3D environments.

The clinical relevance of MEA technology is vast, with applications in neurological disease modeling, high-throughput drug screening, and even brain–computer interface development [26]. MEA provides an invaluable platform for studying neurological disorders, offering real-time insights into disease progression and the effects of therapeutic compounds. Furthermore, the integration of MEA with organ-on-a-chip systems could revolutionize disease modeling and drug screening, enabling the simultaneous monitoring of multiple organ systems and providing more accurate, physiologically relevant data. Limitations in both sensitivity and signal stability, especially in long-term recordings and complex environments, still persist. Although some studies have demonstrated the potential of high-throughput MEA for monitoring neural activity in cerebral organoids [27], enhancing the accuracy and stability of signal detection remains a critical focus for advancing this technology.

Different methods have been attempted to solve the above problem in electrophysiological assessment for organoids, and Pt black electroplating on the electrode surface is found to be effective in cardiac recording and electrical stimulation in rodents, which would significantly reduce electrode impedance and increase the contact area between the electrode and neurons, thereby improving the capture efficiency of neural signals [28,29]. As a porous form of platinum, Pt black’s rough and irregular morphology further enhances the interface between the electrode and neural tissue, providing more contact points for neurons. Moreover, Pt black has been widely documented for its excellent biocompatibility with neuronal cultures. Studies have shown that Pt black-coated electrodes promote stable neuronal adhesion and do not exhibit toxicity, supporting the long-term survival and electrical activity of neurons in cell culture [30]. This improvement is particularly vital for monitoring neuronal activities over extended periods, such as in studies assessing the effects of drugs on the electrophysiological activity of cerebral organoids. However, the application of Pt black electroplating on planar MEA for the electrophysiological signal acquisition of cerebral organoids remains largely unexplored.

In this study, the temporal evolution of neural network activities in cerebral organoids is investigated by comparing electrophysiological recordings obtained at two distinct stages of day 40 and day 180. To enhance the stability and sensitivity of these signals, a 32-channel planar MEA featuring Pt black electroplating is developed. Based on this platform, the electrophysiological activities of human iPSC-derived cerebral organoids were systematically analyzed, focusing on spontaneous neural activities and responses to neuroactive drugs. Specifically, the effects of 30 mM potassium chloride (KCl) on neuronal network activities were investigated, and real-time changes in spike rates and depolarization were recorded. By comparing the neural activities recorded at these developmental stages, our study underscores the progressive maturation of neural networks in cerebral organoids, emphasizing the significance of electrophysiological assessment in understanding this developmental trajectory. Furthermore, the observed robust response to KCl stimulation in mature networks serves as an indicator of cerebral organoid maturity, highlighting this model’s potential for drug screening applications. This work not only confirms the functional maturity of cerebral organoids but also emphasizes the potential of this platform for conducting drug screening and modeling neurological diseases.

## 2. Materials and Methods

### 2.1. Design and Fabrication of the MEA

**Figure 1:**
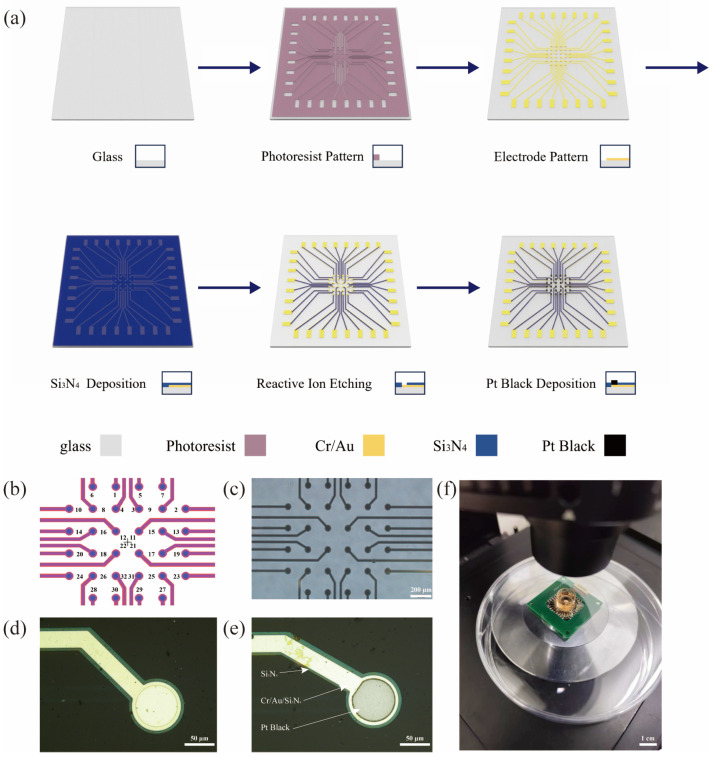
Fabrication process flow and experimental images of the MEA: (**a**) fabrication process flow of the MEA; (**b**) the design layout for MEA positioning sites, where the numbers indicate the corresponding channel positions used for electrophysiological signal acquisition; (**c**) optical microscope image of the fabricated MEA; (**d**) optical microscope image of the recording electrodes before Pt black deposition; (**e**) optical microscope image of the recording electrodes after Pt black deposition; (**f**) photograph of the assembled MEA system.

The manufacturing process of the MEA is illustrated in Figure 1a. It consists of the following steps: The process began with the cleaning of a 300-micron-thick glass wafer using acetone, alcohol, and water, successively. Then, a 50 W oxygen plasma treatment was performed on the wafer for 1 min. The LOR5B photoresist was spin-coated at 4000 rpm and baked at 150 °C for 3 min. After cooling to room temperature, AZ5206 photoresist was immediately spin-coated at 4000 rpm, followed by a 2 min bake at 100 °C. The photoresist was then exposed to 365 nm UV light at a dose of 100 mJ/cm^2^, and the pattern of the electrode point array, wires, and contact pads was developed using AZ 300MIF developer for 20 s. A 5/50 nm thick chromium–gold (Cr/Au) layer was deposited on the wafer using magnetron sputtering, followed by a lift-off process in NMP to define the metal patterns. Next, a 550 nm thick silicon nitride (Si_3_N_4_) insulating layer was deposited over the substrate using the plasma-enhanced chemical vapor deposition (PECVD) method at 130 °C. AZ5214 photoresist was then spin-coated at 4000 rpm and baked at 100 °C for 3 min. The wafer was exposed to 365 nm UV light at a dose of 40 mJ/cm^2^, followed by a reverse bake at 100 °C for 2 min. A flood exposure to 365 nm UV light at 650 mJ/cm^2^ was subsequently performed, and the AZ 300MIF developer was used for 22 s to form the protective layer. Finally, reactive ion etching (RIE) was conducted using CF_4_, argon, and oxygen gases at 180 W for approximately 3 min to expose the electrode points and contact pads on the MEA. The design layout of the 32-channel planar MEA is shown in Figure 1b. Each electrode has a diameter of 50 μm, with a 200 μm inter-electrode spacing, covering a total area of 1 mm^2^ with 32 channels. These design parameters were chosen to strike a balance between spatial resolution and sensitivity, optimizing the capture of both local and network-level neuronal activity in the cerebral organoid. An optical microscope image of the fabricated MEA (Figure 1c) confirms the accurate implementation of this layout, demonstrating the precise alignment of electrode points and contact pads.

### 2.2. Cell Culture

Human induced pluripotent stem cells (iPSCs) sourced from the National Collection of Authenticated Cell Cultures in Shanghai, China (specifically DYR0100) were utilized to facilitate the differentiation into cerebral lineages. The process of inducing cerebral organoids was conducted using the STEMdiff™ Cerebral Organoid Kit (Catalog# 08,570 and 08571) available from STEMCELL Technologies in Vancouver, British Columbia, Canada, following the established protocol meticulously outlined by the manufacturer. To initiate the differentiation process, the iPSCs were initially cultured to form embryoid bodies (EBs) at a density of 9000 cells per well within a 96-well low-attachment U-bottom plate, commencing on day 0. To promote the formation of EBs, further additions of 100 µL of EB formation medium were made to each well on day 2 and day 4. The actual induction phase began on day 5. By day 7, with the observation of dense centers and radial translucent bands within the EBs, the next step involved embedding the EBs in Matrigel (354277, sourced from Corning, NY, USA) and placing them on a shaker while introducing an expansion medium. On day 10, an assessment of the expansion was conducted; if a significant neuroepithelium formation was noted, identifiable by the budding on the surface of the organoids, the medium was then substituted with the maturation medium. Throughout the maturation phase, media exchanges were performed every two days, and the culture was sustained on a shaker for a minimum of 10 days to ensure appropriate development.

### 2.3. Assembly of the MEA System

The MEA system consists of three distinct layers, as shown in Figure S1a. The top layer is a 12 mm-diameter glass ring serving as a culture chamber. The middle layer is a printed circuit board (PCB) that connects to the underlying MEA, ensuring proper electrical signal transmission (Figure S1c). This PCB has connectors compatible with external amplifiers used for electrophysiological data acquisition. The bottom layer consists of a custom-designed 32-channel planar MEA to detect and record the electrical signals. The three layers are bonded together using polydimethylsiloxane (PDMS), which is applied carefully along the edges to seal the structure.

### 2.4. Pt Black Electrochemical Deposition

The electrochemical deposition of Pt black was carried out using the IM6ex electrochemical workstation (ZAHNER elektrik, Kronach, Germany), along with THALES-XT software (version 3.1.2) for current-controlled electrodeposition. A two-electrode configuration was adopted where the Pt wire served as the anodic electrode, and the homemade MEA was the cathode electrode. The precursor solution for deposition was 0.8 wt% chloroplatinic acid. A current density of 0.5 mA/cm^2^ was applied for 5 to 8 min to achieve Pt black deposition onto the surface of the homemade MEA. The optical microscope images of the microelectrode MEA before and after Pt black deposition are shown in Figure 1d and 1e, respectively.

### 2.5. Electrochemical Impedance

Electrochemical impedance spectroscopy studies were performed with an impedance measurement unit (IM6ex, ZAHNER elektrik, Germany). A standard three-electrode configuration was used for the electrochemical impedance measurements, wherein the homemade MEA, a Pt wire electrode and a standard Ag/AgCl electrode are employed as the working electrode, the counter electrode and the reference electrode, respectively. To ensure statistical validity, no fewer than three frequency sweeps were conducted for each assessment with frequencies ranging from 1 MHz to 1 Hz. All experiments were carried out in a 1× PBS solution at a temperature of 25 °C.

### 2.6. Sterilization Procedures

The MEA system underwent a sterilization process to ensure contamination-free operation. Initially, the entire assembly, comprising the glass ring, PCB, and MEA, was subjected to guarantee thorough decontamination. Subsequently, the system was exposed to ultraviolet (UV) light for 30 min to eliminate any residual surface contaminants. Prior to use, the glass ring chamber is filled with DMEM/F12 medium and incubated at 37 °C for 30 min to establish a stable environment for the cerebral organoid experiments.

### 2.7. Electrophysiological Recordings

Electrophysiological activities from the organoid samples were recorded using the Blackrock CerePlex Direct voltage amplifier connected to a 32-channel Blackrock μDigital headstage. The connection between the amplifier and the equipment was established through a custom-designed PCB. To minimize electrical noise, the culture medium of the organoid samples was grounded. A sampling rate of 30,000 samples per second was utilized to ensure high-resolution data collection. Furthermore, a bandpass filter (Butterworth, 4th order) was applied to meet the analysis requirements.

### 2.8. Drug Testing

Cerebral organoids were cultured in a neural maturation medium, and 30 mM KCl was utilized in the drug testing. Baseline recordings were obtained both before and after the administration of drugs. The drugs were introduced into the culture system via a micropipette. After each test, the samples were rinsed for three times with DPBS to eliminate any residual compounds. Electrophysiological recordings were conducted over a 5 min period after KCl administration. For consistency, subsequent analysis focused on the spike rate during this initial minute following stimulation, which was compared to baseline conditions. The spike rate was quantified as the number of spikes detected within this 1 min window.

### 2.9. Statistical Analysis

All the data are presented as the means ± standard deviations for at least three repeats. The data were subjected to statistical analysis using a student’s *t*-test. *p* < 0.05 was considered statistically significant.

## 3. Results

### 3.1. Impedance Evaluation of the MEA

**Figure 2:**
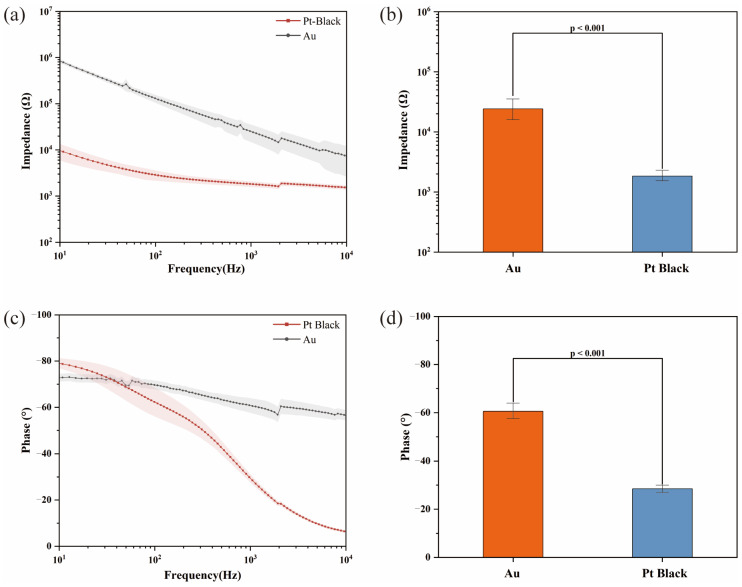
Modification and characterization of the MEA: (**a**) electrochemical impedance spectroscopy (EIS) of the recording MEA sites before modification with Pt black; (**b**) an average impedance of 32 sites at 1 kHz decreased from 24,117.35 ± 1770.29 (SE) Ω to 1840.38 ± 43.90 (SE) Ω (t = 12.58, *p* < 0.001); (**c**) phase distribution characterization of the recording sites before modification with Pt black; (**d**) average phase of 32 sites at 1 kHz changed from −60.55 ± 0.57 (SE)° to −28.50 ± 1.75 (SE)° (t = 49.07, *p* < 0.001). Statistical analysis was performed using paired *t*-test.

Electrochemical impedance measurements were carried out to evaluate the performance of the 32-channel planar MEA before and after the deposition of Pt black. Figure 2a illustrates the impedance magnitude before and after Pt black deposition. Figure 2b demonstrates that at a frequency of 1 kHz, the impedance significantly decreased by more than tenfold following Pt black deposition, from 24,117.35 ± 1770.29 (SE, Standard Error) Ω to 1840.38 ± 43.90 (SE) Ω (t = 12.58, *p* < 0.001). This reduction is attributed to the increased effective surface area provided by the Pt black, which enhances charge transfer at the electrode interface, improving the electrode’s sensitivity for neural signal detection. Furthermore, Figure 2c shows the phase response before and after Pt black deposition. A notable shift in the phase was observed at low frequencies, indicating enhanced capacitive coupling following Pt black modification. The phase shifted from −60.55 ± 0.57 (SE)° to −28.50 ± 1.75 (SE)° (t = 49.07, *p* < 0.001), further confirming the improvement in electrode performance post-modification, as illustrated in Figure 2d.

### 3.2. Organoid Development

**Figure 3:**
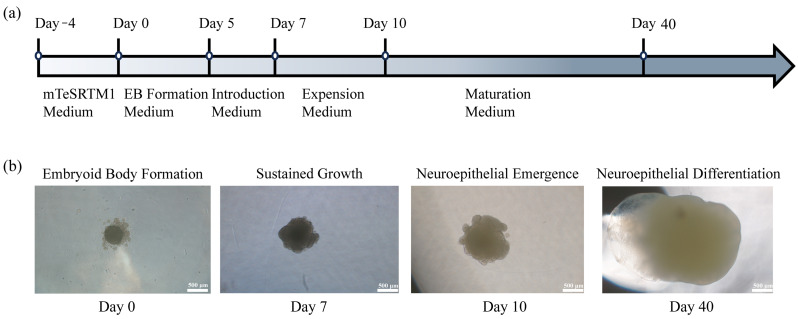
Formation of human cerebral organoids using human induced pluripotent stem cell (hiPSC): (**a**) timeline for cerebral organoids differentiation from hiPSC; (**b**) optical microscopic observation of cerebral organoids development at distinct differentiation stages.

The development of cerebral organoids from hiPSCs was achieved using a stepwise differentiation protocol facilitated by the STEMdiff™ Cerebral Organoid Kit, and the timeline for cerebral organoid differentiation from hiPSC is shown in Figure 3a. The process began with forming EBs on day 0, cultured in low-attachment U-bottom plates to promote aggregation and spherical morphology. By day 5, the EBs exhibited increased cellular density and formed organized structures, marking the onset of neural induction. On day 7, the EBs demonstrated distinctive morphological changes, including dense centers and peripheral radial translucent bands, indicative of neural differentiation. These structures were embedded in Matrigel to provide a 3D extracellular matrix environment supporting further development. Organoids were then cultured on an orbital shaker in an expansion medium to facilitate uniform nutrient and oxygen distribution. By day 10, neuroepithelial formations with budding structures were observed on the organoid surfaces, signaling the successful establishment of neural lineage differentiation. At this stage, the medium was transitioned to a maturation medium to support the development of complex neural architecture. Medium exchanges were conducted every two days, and the organoids were maintained on the shaker to ensure consistent growth. The maturation phase extended for at least 30 additional days, during which the cerebral organoids acquired advanced structural features, including cortical-like regions. This approach generated organoids capable of recapitulating early human brain development, providing a suitable model for downstream analysis and experimental applications (Figure 3b).

**Figure 4:**
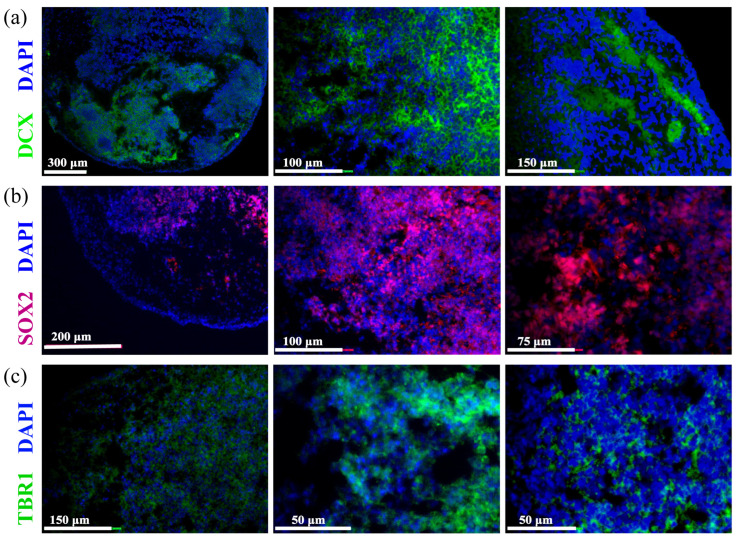
Immunofluorescence characterizations of cerebral organoids on day 70 of differentiation: (**a**) DCX expression (green) marking neuronal precursor cells, with DAPI (blue) counterstaining for nuclear visualization; (**b**) SOX2 expression (red) indicates neural progenitor cells, with DAPI (blue) counterstaining highlighting cell nuclei in the peripheral regions of the organoids; (**c**) TBR1 expression (green) in differentiated cortical neurons, with DAPI (blue), counterstaining to visualize cell nuclei, highlighting deep-layer cortical neurons within the organoid structure.

To further characterize the neural differentiation and maturity of the cerebral organoids, immunofluorescence staining for symbolic neuronal proteins was conducted at approximately day 70, representing an intermediate time point to capture the structural characteristics during the transition from early-stage progenitor activity to more mature neural organization. The presence of early-stage differentiating neurons was assessed using the expression of neuronal precursor marker, the Doublecortin (DCX) protein, which showed green fluorescence indicating the presence of early-stage differentiating neurons (Figure 4a, red). Neural progenitor cells were identified by the expression of the SRY-box transcription factor 2 (SOX2) protein, which was primarily localized in the peripheral regions of the organoids, reflecting the undifferentiated stem cell population (Figure 4b, purple). The presence of differentiated cortical neurons was assessed by the expression of T-box brain transcription factor 1 (TBR1), which was detected in the deeper layers of the organoids, indicating the successful differentiation of cortical-like neurons (Figure 4c, green). These findings confirm the establishment of organized neural structures within the cerebral organoids and highlight their applicability for subsequent electrophysiological assessment using the MEA platform, as well as their potential for studying complex brain development and disease models.

### 3.3. Electrophysiological Activity Recordings

**Figure 5:**
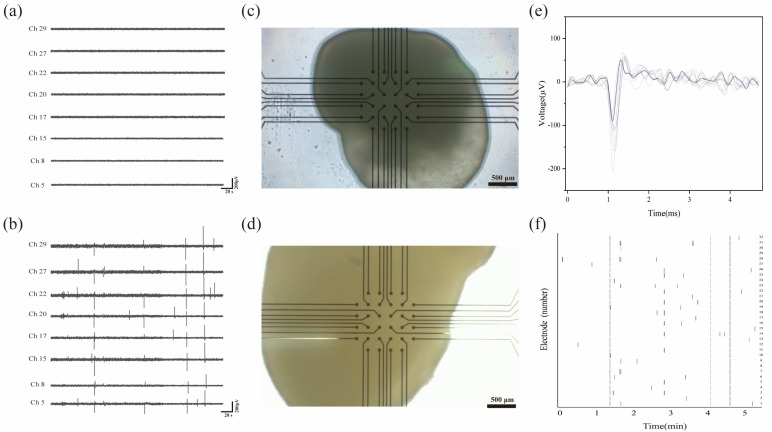
MEA recordings of cerebral organoid electrical activity: (**a**) representative electrophysiological recording from cerebral organoids on day 40; (**b**) representative electrophysiological recording from cerebral organoids on day 180; (**c**) optical microscopy image of cerebral organoids cultured on MEA systems on day 40; (**d**) optical microscopy image of cerebral organoids cultured on MEA systems on day 180; (**e**) representative overlaid spike waveforms captured from active channels on day 180; (**f**) raster plot illustrating spike distribution across active channels within 5 min on day 180.

Cerebral organoids differentiated for 40 and 180 days were transferred to the custom-designed planar MEA for electrophysiological recordings. Each recording was performed for 5 min, with a sampling rate of 30 kHz. During signal acquisition, a 250 Hz low-pass filter was applied to preprocess the data and suppress high-frequency noise. Figure 5a shows that on day 40, no significant electrophysiological signals were detected, indicating that the neural networks within the organoids had not yet matured to a functional stage. In contrast, on day 180 (Figure 5b), spontaneous neural activity was successfully recorded, reflecting the functional maturation of neural networks. These findings are consistent with previous studies demonstrating that spontaneous neural activity arises in cerebral organoids as their neural networks mature during differentiation [27]. To account for any batch-to-batch variability, we ensured the consistency of measurements by using organoids from the same batch, and the data were analyzed statistically to confirm that differences observed between the D40 and the D180 stages were significant. Spiking activity was detected across multiple channels, reflecting the functional electrophysiological properties of the organoids. Meanwhile, bright-field imaging in Figure 5c,d suggests that compared with day 40, the cerebral organoid on day 180 had grown significantly in size. On day 180, representative extracellular waveforms demonstrated characteristic spike shapes (Figure 5e), with amplitudes ranging from −200 μV to 50 μV and durations of approximately 4 ms. Raster plots of active electrodes revealed isolated spikes and propagating events indicative of neuronal connectivity (Figure 5f). The average spike rate across 32 channels was 0.178 spikes per second, highlighting stable neural firing patterns.

These findings underscore the maturation of neural networks in cerebral organoids at later developmental stages and validate the MEA platform’s capability for stable, real-time monitoring of spontaneous electrophysiological activity.

### 3.4. Drug Effects on Cerebral Organoids

**Figure 6:**
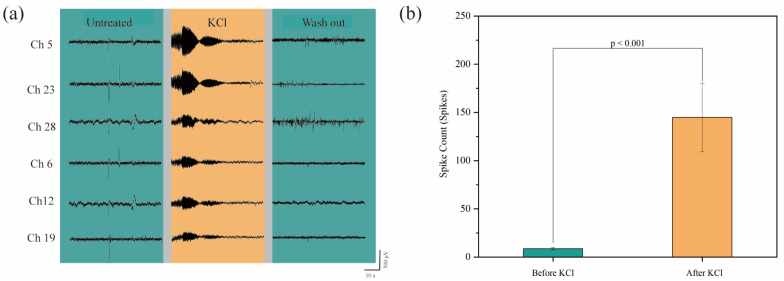
Pharmacological response of cerebral organoids at late stage: (**a**) raw voltage traces from six representative channels, showing the electrophysiological response under untreated, 30 mM KCl stimulation, and washout conditions; (**b**) spike rate comparison across all 32 channels within 1 min before and after KCl administration demonstrates a significant increase in neural activity, confirming the robust activation of the neural network.

On day 180, we introduced 30 mM KCl stimulation to further investigate the pharmacological response of the mature neural networks. This stimulation induced a significant increase in spike rate, demonstrating robust depolarization and activation of the neural network, further supporting the maturation of the organoid model. To ensure that the recorded signals truly reflect neuronal responses rather than impedance changes caused by KCl on the electrode surface, we first assessed baseline stability by recording spontaneous activity from cerebral organoids prior to KCl stimulation. This provided a reliable reference for distinguishing neuronal activity from potential artifacts. We also observed a clear increase in firing rate across multiple channels, with distinct and heterogeneous spike patterns, which are characteristic of neuronal depolarization rather than uniform fluctuations caused by impedance changes. To minimize potential noise from organoid movement or displacement during recording, we ensured that the organoids were firmly attached to the culture substrate, and the culture medium was carefully stabilized to avoid flow-induced disturbances. In Figure 6a, we present representative raw voltage traces from six selected channels, illustrating the electrophysiological response under three conditions: untreated, 30 mM KCl stimulation, and washout. Prior to KCl administration, the average spike rate across all channels was 0.133 spikes per second. After introducing KCl, the spike rate significantly increased to 2.4 spikes per second, indicating a robust activation of the neural network within the organoids. Statistical analysis using a paired *t*-test (assuming equal variances) revealed a significant difference between the pre- and post-KCl spike rates, with a t-statistic of 3.825 (Figure 6b, *p* < 0.001). These results demonstrate the functional maturity of cerebral organoids on day 180 and highlight the responsiveness of mature networks to KCl stimulation. The significant increase in neural activity induced by KCl underscores the potential of this system for studying network excitability and exploring its applications in pharmacological research. In Figure S4, raw voltage traces from three selected channels on day 40 and day 180 show no response to KCl on day 40, further supporting the maturation of the neural network by day 180.

## 4. Discussion

This study presents the application of a custom planar MEA enhanced with Pt black electroplating to assess the electrophysiological activity in human pluripotent stem cell-derived cerebral organoids. We observed spontaneous neural activity and pharmacological responses through real-time electrophysiological monitoring, highlighting the neural connectivity and functional maturity achieved in these organoids. The Pt black coating significantly improved signal sensitivity and stability by reducing impedance, enabling reliable and high-resolution neuro electrical recordings. Compared to prior studies, our findings demonstrate the effectiveness of advanced electrode modifications, aligning with advancements in MEA technologies aimed at optimizing long-term neural recordings (Table 1). Our study further revealed significant insights into the response of cerebral organoids to pharmacological stimuli. The administration of 30 mM KCl induced a substantial increase in spike rate, indicative of enhanced neural activity due to depolarization. This finding aligns with typical neuronal behavior observed in in vitro models and provides strong evidence of the functional maturity of the cerebral organoid [11,31]. These pharmacological responses not only validate the organoid model’s ability to mimic physiological changes but also highlight its potential for modeling neurodevelopmental processes and studying disease mechanisms. The consistent neural firing and responsiveness to external stimuli position cerebral organoids as a promising platform for exploring dynamic neural network activity and testing pharmacological compounds. Although molecular validation via qPCR analysis was not included in this study, incorporating such analyses in future work would complement the electrophysiological data by providing insights into neural differentiation and maturation at the molecular level. Previous studies have shown that combining qPCR with electrophysiological measurements enhances the understanding of functional maturation in organoid models [32,33]. Future integration of qPCR could further validate the progression of neural network formation and maturation, strengthening our findings.

Despite these promising findings, several limitations of this study still need to be addressed. Although the planar MEA configuration was effective for initial electrophysiological analysis, it lacked the spatial resolution to fully characterize the complex 3D interactions within the organoids. Despite these promising findings, several limitations of this study still need to be addressed. Although the planar MEA configuration was effective for initial electrophysiological analysis, it lacked the spatial resolution to fully characterize the complex 3D interactions within the organoids. Moreover, the analysis of network connectivity and burst patterns, which are essential for assessing the functional maturation of organoid networks, remains challenging due to these spatial and temporal constraints. The current system has provided valuable insights into neural network activity over extended periods, which is an important step toward evaluating the maturation of cerebral organoid networks. However, maintaining consistent, high-quality signals over long durations remains a challenge and an area for further optimization. Future studies will focus on improving the sustainability and robustness of the electrophysiological signals to better capture the long-term development of organoid networks and their responses to pharmacological stimuli. Such advancements will provide deeper insights into the evolution and maturation of cerebral organoids and help enhance their potential for neurological disease modeling and drug screening. These limitations may hinder the accurate representation of in vivo-like neural dynamics. To address these issues, future studies should consider extending the recording duration to explore long-term neural development and stability, providing deeper insights into the maturation processes of cerebral organoids. Enhancing the spatial resolution of MEA designs would enable more precise mapping of fine-scale neural network interactions. Moreover, integrating advanced computational methods, such as machine learning algorithms, could facilitate deeper analysis of complex neural activity patterns and support applications in brain–computer interfaces and biohybrid systems [34,35]. Finally, combining cerebral organoid models with other technologies, such as 3D electrode arrays or organ-on-a-chip platforms, may provide a more comprehensive understanding of organoid behavior and improve their translational potential for neurological research [36,37].

In conclusion, this study establishes a foundational approach for the electrophysiological investigation of cerebral organoids, providing a sensitive and stable platform for neural activity analysis. By addressing current limitations and exploring new technological integrations, cerebral organoids hold great promise as models for understanding human brain function, studying neurodevelopmental disorders, and advancing drug discovery efforts.

**Table 1:** Comparison of electrophysiological techniques: custom Pt black MEA, CMOS–MEA, flexible MEA, and traditional planar MEA.

Technology	Number of Channels	Applications	Impedance (1 kHz)	Reference
CMOS–MEA	4096	Neuronal networks	300 kΩ	[38]
Flexible MEA	16	In vivo brain tissue	60 kΩ	[39]
Planar MEA	16	Astrocytic cultures	Not Available	[40]
Custom MEA	32	Organoids	2 kΩ	This work

## 5. Conclusions

This study successfully demonstrated the use of a custom 32-channel planar MEA with Pt black coating to capture and analyze the electrophysiological activity of human pluripotent stem cell-derived cerebral organoids. The enhanced MEA significantly improved signal sensitivity and stability, enabling reliable detection of spontaneous neural activity and pharmacological responses. By comparing recordings from cerebral organoids on day 40 and day 180, we observed a clear progression in neural network maturation. While no significant electrophysiological activity was detected on day 40, robust spontaneous spiking activity on day 180 indicated the development of functional neural networks. These results underscore the temporal evolution of neural network activity during organoid maturation. The observed increase in spike rates following KCl administration further validated the responsiveness and functional maturity of the cerebral organoids on day 180, demonstrating the utility of KCl-induced depolarization as a method to probe network excitability. This highlights the potential of this platform for investigating dynamic neural network activity and pharmacological responses, offering a valuable tool for neurodevelopmental research and drug testing. These findings emphasize the utility of MEA technology for investigating in vitro neural network dynamics and the applicability of cerebral organoids in neurodevelopmental research and pharmacological testing. This research also presents a new experimental platform that combines advanced electrophysiological monitoring with the study of 3D organoid models, offering new insights into neural network function, drug response, and disease modeling. However, while the planar MEA configuration effectively captures surface-level neural activity, it cannot fully represent the 3D electrophysiological dynamics of cerebral organoids, limiting its capacity to reflect the spatial complexity of neural networks. Moreover, the relative positioning of organoids and the planar MEA is not fixed. Over time, organoids may drift within the culture system, leading to variability in recording sites and potential inconsistencies in region-specific data during longitudinal studies. To address these challenges, future research should focus on stabilizing the organoid–MEA interface to ensure consistent recording conditions. Improvements in spatial resolution, electrode coverage, and advanced computational approaches, such as machine learning for signal analysis, are also critical. These advancements could enhance the accuracy and reliability of electrophysiological studies, strengthening the role of cerebral organoids in modeling complex neural networks, studying neurodevelopmental disorders, and advancing drug screening applications.

Future research should address these limitations by extending recording durations to study long-term organoid development, improving the spatial resolution of electrode arrays, and integrating advanced computational approaches such as machine learning. Such advancements could expand the applications of cerebral organoids in modeling neurodevelopmental disorders, exploring brain–computer interfaces, and developing biohybrid systems, ultimately enhancing their role in translational neuroscience research.

## Data Availability

The data presented in this study are available upon request from the corresponding author.

## Supplementary Materials

The following supporting information can be downloaded at: https://www.mdpi.com/article/10.3390/bios15030173/s1, Figure S1. Schematic diagram of MEA system: (a) schematic diagram of the three-layer structure of the MEA system; (b) schematic diagram of the signal output interface (A79024-001); (c) PCB layout diagram; Figure S2. SEM images of Pt black electroplated surface: (a) SEM image of the electrode surface after Pt black electroplating, showing increased surface roughness; (b) SEM image of the electrode surface before Pt black electroplating, exhibiting a smoother surface; Figure S3. Statistical comparison of Diameter at D40 and D180 of cerebral organoid development; Figure S4. Raw voltage traces from three channels following 30 mM KCl stimulation at D40 and D180 of cerebral organoids development.

## References

[1] Tsytsarev V., Sopova J.V., Leonova E.I., Inyushin M., Markina A.A., Chirinskaite A.V., Volnova A.B. (2024). Neurophotonic Methods in Approach to in Vivo Animal Epileptic Models: Advantages and Limitations. Epilepsia.

[2] De Vitis E., Stanzione A., Romano A., Quattrini A., Gigli G., Moroni L., Gervaso F., Polini A. (2024). The Evolution of Technology-Driven In Vitro Models for Neurodegenerative Diseases. Adv. Sci..

[3] Osaki T., Shin Y., Sivathanu V., Campisi M., Kamm R.D. (2018). In Vitro Microfluidic Models for Neurodegenerative Disorders. Adv. Healthc. Mater..

[4] Park Y., Franz C.K., Ryu H., Luan H., Cotton K.Y., Kim J.U., Chung T.S., Zhao S., Vazquez-Guardado A., Yang D.S. (2021). Three-Dimensional, Multifunctional Neural Interfaces for Cortical Spheroids and Engineered Assembloids. Sci. Adv..

[5] Sloan S.A., Andersen J., Pașca A.M., Birey F., Pașca S.P. (2018). Generation and Assembly of Human Brain Region–Specific Three-Dimensional Cultures. Nat. Protoc..

[6] Paşca A.M., Sloan S.A., Clarke L.E., Tian Y., Makinson C.D., Huber N., Kim C.H., Park J.-Y., O’Rourke N.A., Nguyen K.D. (2015). Functional Cortical Neurons and Astrocytes from Human Pluripotent Stem Cells in 3D Culture. Nat. Methods.

[7] Anderson W.A., Bosak A., Hogberg H.T., Hartung T., Moore M.J. (2021). Advances in 3D Neuronal Microphysiological Systems: Towards a Functional Nervous System on a Chip. Vitro Cell Dev. Biol.-Anim..

[8] Di Lullo E., Kriegstein A.R. (2017). The Use of Brain Organoids to Investigate Neural Development and Disease. Nat. Rev. Neurosci..

[9] Grenier K., Kao J., Diamandis P. (2020). Three-Dimensional Modeling of Human Neurodegeneration: Brain Organoids Coming of Age. Mol. Psychiatry.

[10] Eichmüller O.L., Knoblich J.A. (2022). Human Cerebral Organoids—A New Tool for Clinical Neurology Research. Nat. Rev. Neurol..

[11] Lancaster M.A., Renner M., Martin C.-A., Wenzel D., Bicknell L.S., Hurles M.E., Homfray T., Penninger J.M., Jackson A.P., Knoblich J.A. (2013). Cerebral Organoids Model Human Brain Development and Microcephaly. Nature.

[12] Bershteyn M., Kriegstein A.R. (2013). Cerebral Organoids in a Dish: Progress and Prospects. Cell.

[13] McCracken K.W., Catá E.M., Crawford C.M., Sinagoga K.L., Schumacher M., Rockich B.E., Tsai Y.-H., Mayhew C.N., Spence J.R., Zavros Y. (2014). Modelling Human Development and Disease in Pluripotent Stem-Cell-Derived Gastric Organoids. Nature.

[14] Babu H.W.S., Kumar S.M., Kaur H., Iyer M., Vellingiri B. (2024). Midbrain Organoids for Parkinson’s Disease (PD)—A Powerful Tool to Understand the Disease Pathogenesis. Life Sci..

[15] Kim H., Park H.J., Choi H., Chang Y., Park H., Shin J., Kim J., Lengner C.J., Lee Y.K., Kim J. (2019). Modeling G2019S-LRRK2 Sporadic Parkinson’s Disease in 3D Midbrain Organoids. Stem Cell Rep..

[16] Chang Y., Kim J., Park H., Choi H., Kim J. (2020). Modelling Neurodegenerative Diseases with 3D Brain Organoids. Biol. Rev..

[17] Andrews M.G., Kriegstein A.R. (2022). Challenges of Organoid Research. Annu. Rev. Neurosci..

[18] Quadrato G., Brown J., Arlotta P. (2016). The Promises and Challenges of Human Brain Organoids as Models of Neuropsychiatric Disease. Nat. Med..

[19] Urrestizala-Arenaza N., Cerchio S., Cavaliere F., Magliaro C. (2024). Limitations of Human Brain Organoids to Study Neurodegenerative Diseases: A Manual to Survive. Front. Cell. Neurosci..

[20] Passaro A.P., Stice S.L. (2021). Electrophysiological Analysis of Brain Organoids: Current Approaches and Advancements. Front. Neurosci..

[21] Trujillo C.A., Gao R., Negraes P.D., Gu J., Buchanan J., Preissl S., Wang A., Wu W., Haddad G.G., Chaim I.A. (2019). Complex Oscillatory Waves Emerging from Cortical Organoids Model Early Human Brain Network Development. Cell Stem Cell.

[22] Gao J., Zhang H., Xiong P., Yan X., Liao C., Jiang G. (2020). Application of Electrophysiological Technique in Toxicological Study: From Manual to Automated Patch-Clamp Recording. TrAC Trends Anal. Chem..

[23] Sucher N.J., Deitcher D.L. (1995). PCR and Patch-Clamp Analysis of Single Neurons. Neuron.

[24] Wu H., Gemes G., Hogan Q.H., Penna A., Constantin B. (2018). Recording SOCE Activity in Neurons by Patch-Clamp Electrophysiology and Microfluorometric Calcium Imaging. The CRAC Channel.

[25] Formozov A., Chini M., Dieter A., Yang W., Pöpplau J.A., Hanganu-Opatz I.L., Wiegert J.S. (2022). Calcium Imaging and Electrophysiology of Hippocampal Activity under Anesthesia and Natural Sleep in Mice. Sci. Data.

[26] Liu Y., Xu S., Yang Y., Zhang K., He E., Liang W., Luo J., Wu Y., Cai X. (2023). Nanomaterial-Based Microelectrode Arrays for in Vitro Bidirectional Brain–Computer Interfaces: A Review. Microsyst. Nanoeng..

[27] Yakoub A.M. (2019). Cerebral Organoids Exhibit Mature Neurons and Astrocytes and Recapitulate Electrophysiological Activity of the Human Brain. Neural Regen. Res..

[28] Sunwoo S., Han S.I., Kang H., Cho Y.S., Jung D., Lim C., Lim C., Cha M., Lee S., Hyeon T. (2020). Stretchable Low-Impedance Nanocomposite Comprised of Ag–Au Core–Shell Nanowires and Pt Black for Epicardial Recording and Stimulation. Adv. Mater. Technol..

[29] Stanca S.E., Hänschke F., Ihring A., Zieger G., Dellith J., Kessler E., Meyer H.-G. (2017). Chemical and Electrochemical Synthesis of Platinum Black. Sci. Rep..

[30] Lee Y.J., Kim H., Kang J.Y., Do S.H., Lee S.H. (2017). Biofunctionalization of Nerve Interface via Biocompatible Polymer-Roughened Pt Black on Cuff Electrode for Chronic Recording. Adv. Healthc. Mater..

[31] Giandomenico S.L., Sutcliffe M., Lancaster M.A. (2021). Generation and Long-Term Culture of Advanced Cerebral Organoids for Studying Later Stages of Neural Development. Nat. Protoc..

[32] Gomes A.R., Fernandes T.G., Vaz S.H., Silva T.P., Bekman E.P., Xapelli S., Duarte S., Ghazvini M., Gribnau J., Muotri A.R. (2020). Modeling Rett Syndrome With Human Patient-Specific Forebrain Organoids. Front. Cell Dev. Biol..

[33] De Kleijn K.M.A., Zuure W.A., Straasheijm K.R., Martens M.B., Avramut M.C., Koning R.I., Martens G.J.M. (2023). Human Cortical Spheroids with a High Diversity of Innately Developing Brain Cell Types. Stem Cell Res. Ther..

[34] Livezey J.A., Glaser J.I. (2021). Deep Learning Approaches for Neural Decoding across Architectures and Recording Modalities. Brief. Bioinform..

[35] Shukla S., Comerci C.J., Süel G.M., Jahed Z. (2025). Bioelectronic Tools for Understanding the Universal Language of Electrical Signaling across Species and Kingdoms. Biosens. Bioelectron..

[36] Song J., Jeong H.E., Choi A., Kim H.N. (2024). Monitoring of Electrophysiological Functions in Brain-on-a-chip and Brain Organoids. Adv. NanoBiomed Res..

[37] Aydogmus H., Hu M., Ivancevic L., Frimat J.-P., van den Maagdenberg A.M.J.M., Sarro P.M., Mastrangeli M. (2023). An Organ-on-Chip Device with Integrated Charge Sensors and Recording Microelectrodes. Sci. Rep..

[38] Abbott J., Ye T., Krenek K., Gertner R.S., Wu W., Jung H.S., Ham D., Park H. (2020). Extracellular Recording of Direct Synaptic Signals with a CMOS-Nanoelectrode Array. Lab Chip.

[39] Zhou C., Tian Y., Li G., Ye Y., Gao L., Li J., Liu Z., Su H., Lu Y., Li M. (2024). Through-Polymer, via Technology-Enabled, Flexible, Lightweight, and Integrated Devices for Implantable Neural Probes. Microsyst. Nanoeng..

[40] Kuroda T., Matsuda N., Ishibashi Y., Suzuki I. (2023). Detection of Astrocytic Slow Oscillatory Activity and Response to Seizurogenic Compounds Using Planar Microelectrode Array. Front. Neurosci..

