# Supplementary material for: Non-Invasive and Long-Term Electrophysiological Monitoring Sensors for Cerebral Organoids Differentiation
Source: Biosensors (Basel). 2025 Mar 7;15(3):173. doi: 10.3390/bios15030173 (PMC11940203; doi:10.3390/bios15030173)
Supplement: Supplementary file 1 [file biosensors-15-00173-s001.zip › biosensors-3484229-supplementary.pdf]

This PDF file includes:

Supplementary Figures S1 to S4

The following supplementary figures provide additional data and details supporting the findings presented in the manuscript. These figures include schematics of the MEA system, SEM images of Pt black electroplated electrodes and representative electrophysiological data recorded from cerebral organoids. Together, these materials offer further insight into the experimental setup, the surface characteristics of the electrodes, and the electrophysiological responses.

Supplementary Materials for this manuscript include the following:

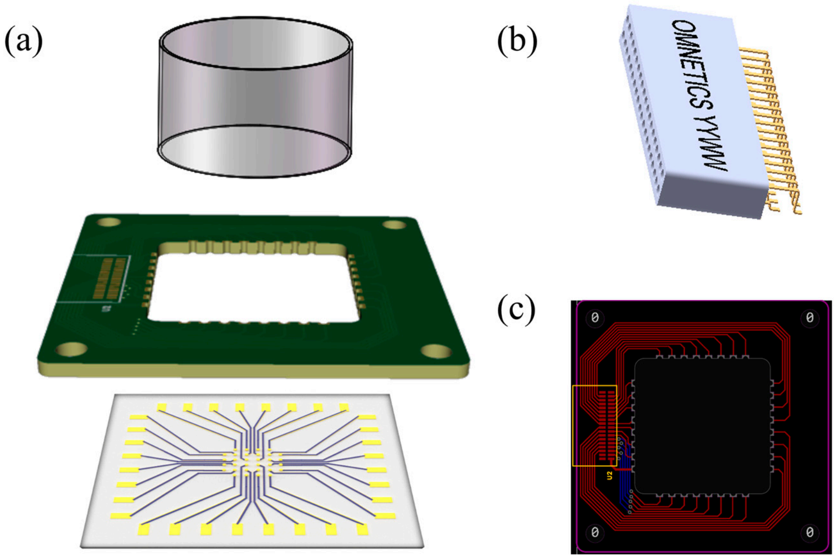

Figure S1. Schematic diagram of MEA system: (a) Schematic diagram of the three-layer structure of the MEA system; (b) Schematic diagram of the signal output interface (A79024-001); (c) PCB layout diagram.

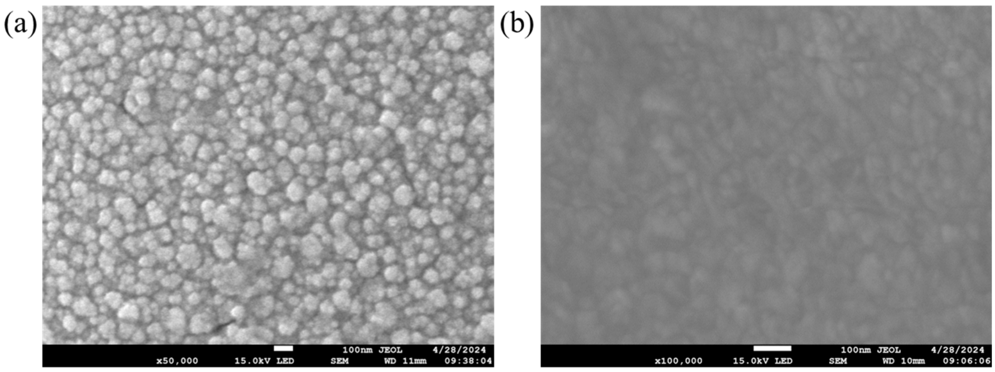

Figure S2. SEM images of Pt black electroplated surface: (a) SEM image of the electrode surface after Pt black electroplating, showing increased surface roughness. (b) SEM image of the electrode surface before Pt black electroplating, exhibiting a smoother surface.

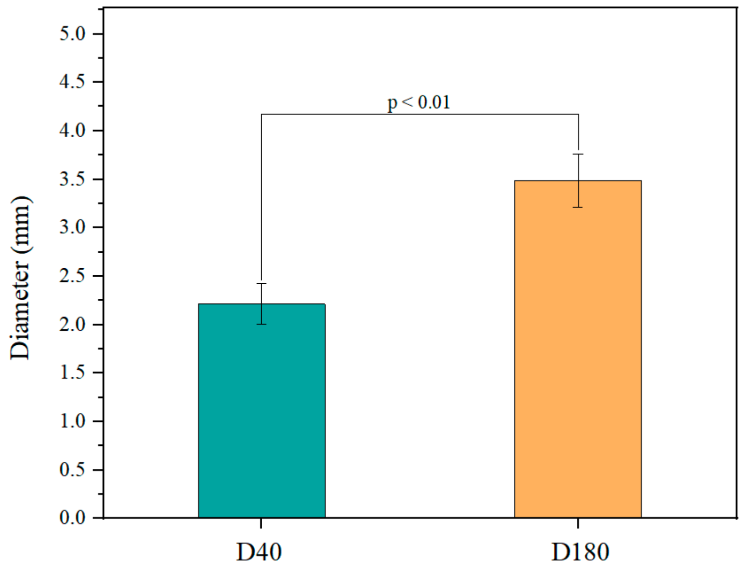

Figure S3. Statistical comparison of Diameter at D40 and D180 of cerebral organoid development.

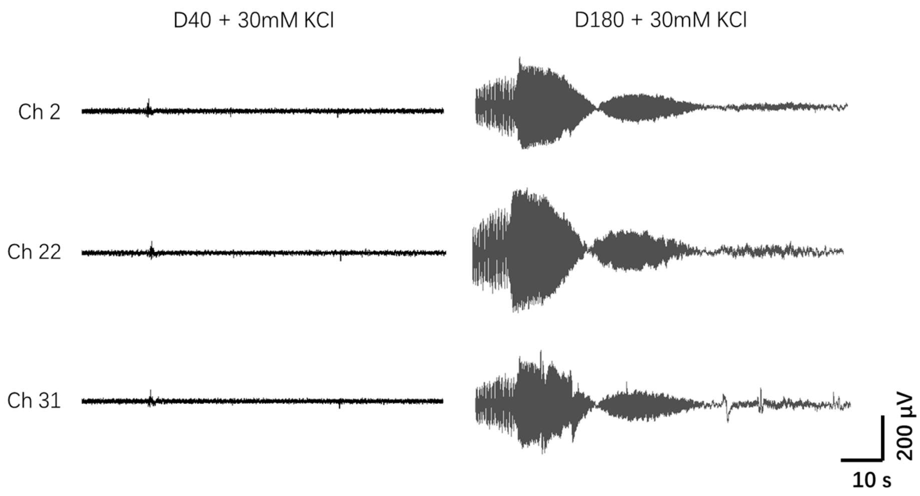

Figure S4. Raw voltage traces from three channels following 30 mM KCl stimulation at D40 and D180 of cerebral organoids development.
